# Conversion of external fixation to internal fixation in a non-acute, reconstructive setting: a case series

**DOI:** 10.1007/s11751-013-0157-8

**Published:** 2013-03-09

**Authors:** T. Monni, F. F. Birkholtz, P. de Lange, C. H. Snyckers

**Affiliations:** 1 Steve Biko Academic Hospital, Pretoria, Gauteng South Africa; 2 Private Practise, Netcare Unitas Hospital, Pretoria, Gauteng South Africa; 3 Department of Orthopaedics, University of Pretoria, Pretoria, Gauteng South Africa

**Keywords:** External fixation, Internal fixation, Conversion, Limb reconstruction, Scoring system, Consolidation phase

## Abstract

The aim of the study is to determine the outcomes in patients who underwent conversion from an external fixator to an internal fixation device. This is a retrospective review of 18 patients (24 limbs) who underwent conversion from external to internal fixation. The patients had external fixators applied for traumatic bone defects or congenital deformities. Conversion to internal fixation was performed for reasons of patient dissatisfaction with external fixation, pin track sepsis, persistent non-union or refracture. The complexity of cases was graded using Paley’s level of difficulty score. Patients were either converted acutely or delayed. Internal fixation devices were either intramedullary nails or plate and screws. Outcome was regarded as excellent if the patients were fully weight-bearing and pain-free on a mechanically well-aligned limb and without need for further surgery: good if the patient required subsequent surgery to achieve union and poor if irreversible complications occurred. Acute conversions (fixator removal and introduction of internal fixation device at same surgery) were done in 19 limbs and delayed conversion (interval between fixator removal and internal fixation) in 5. In the acute group, 17 limbs (89.4 %) had at least a good outcome, 16 of these limbs had an excellent result. Two limbs (10.6 %) had a poor result and required amputation. Both cases were after acute conversion to intramedullary nails; the original presenting diagnosis was of an infected non-union of the tibia and both had Paley scores above 7. In the delayed conversion group, all limbs (100 %) had at least a good outcome, with 4 limbs (80 %) having an excellent result. The mean external fixator time was 185 days (61–370). Both the cases with poor outcomes had longer external fixation times. This series supports the practice of conversion of external fixation to internal fixation with the majority of patients attaining good results. It identifies that plate devices appear to produce fewer deep sepsis complications, as compared to intramedullary nails, particularly when the original presenting diagnosis is a septic non-union.

## Introduction

Despite the versatility of distraction osteogenesis in limb reconstruction surgery, prolonged external fixation is uncomfortable for the patient and has associated complications [1, 2, 8, 9, 14]. Methods to decrease frame time have been developed; these include lengthening over a nail [3, 4, 7, 11, 15] and lengthening with submuscular plating [5, 6, 12] from which patients have shown improved comfort and recovery of joint range of motion. The risk of combining external and internal fixation is deep infection. This is documented to be 3–15 % [11, 13]. There is no consensus as to which internal fixation method, when used after external fixation, leads to better results.

Rozbruch et al. [16] suggested that the reaming through the regenerate enhances bone healing but was concerned over the infection risk with use of intramedullary nails. He felt it important to pay special attention to the placement of external fixator pins to avoid contact between the nail and the pin sites. His reported deep infection rate was 2.5 %. He went on to investigate the technique of lengthening then plating. He found a decreased frame time but no deep infections [17]. Uysal et al. [10] believed that both the endosteal and periosteal blood supplies are preserved with this technique. However, Rozbruch et al. [17] did note a high incidence of varus deformity.

The literature is limited on the subject of sequential use of internal fixation after external fixation in post-traumatic limb reconstruction and deformity correction. The technique would decrease frame time in the treatment for post-traumatic bone loss and non-unions as well as deformity corrections and prove valuable but has the risk for complications.

## Materials and methods

This is a retrospective case series on 18 patients (24 limbs) who underwent sequential conversion from external to internal fixation in the period 2007–2011. All patients who underwent distraction osteogenesis for traumatic bone loss, sepsis or for the correction of deformities and had internal fixation applied prior to union or regenerate consolidation were included. There were no specific exclusion criteria.

Patients were grouped according to the timing of conversion from external to internal fixation as well the type of internal fixation used. The following groups were defined:

1. The acute conversion group consisted of patients who underwent removal of the external fixator device and insertion of internal fixation at the same surgical procedure. The operation also consisted of debridement of the external fixation pin tracks and careful placement of the internal fixation device with care to avoid contact with the previous external fixation pin sites.
2. The delayed conversion group consisted of patients who underwent separate procedures for removal of external fixation and placement of the internal fixation device. Debridement of external fixation pin tracks was done during the first procedure. Stability in the interval between procedures was achieved by various methods including traction, plaster of Paris and functional braces. This was individualized according to site and stability. This interval varied and the secondary procedure was performed when the surgeon deemed the pin tracks to be healed with no infection.

The internal fixation devices were either intramedullary nails or plates and screws.

An available scoring system to allow for sample description or classification was not identified. We chose to adopt Paley’s level of difficulty score for femoral lengthening in which 11 variables are separately evaluated and include not only host and local factors but also the complexity of correction (Table 1).

**Table 1:** Paley’s level of difficulty score [4]

Points scored	0	1	2	3
Age	5–19	20–29, 0–4	30–50	>50
Complexity of correction of deformity at level of lengthening	None	Angulation >5° <20° Rotation >10° <30° Translation <50 % or change of mechanical axis 1–3 cm	Angulation ≥20° Rotation ≥30° Translation ≥50 % or change of mechanical axis >3 cm	Combination of deformities at one level or multilevel deformity
Other levels of treatment in same bone	None	1 Additional level, mild complexity	1 Additional level, moderate complexity	1 Additional level, severe complexity or ≥addition
Associated tibial lengthening (cm)	None	1–3	3.1–6	>6
Instability of joint	None	Grade I—mild instability: anteroposterior instability of knee with end point. Shenton’s line not broken	Grade II—moderate instability: anteroposterior instability of knee without end point. Shenton’s line broken but reducible	Grade III—fixed subluxation or dislocation
Fixed flexion deformity of knee (°)	0	1–5	6–20	>20
Flexion of knee (°)	>120	100–120	65–99	<65
Osteoarthrosis of joint	None	Marginal osteophytes, subchondral sclerosis	Narrowing of joint space	Loss of joint space (bone on bone)
Quality of bone	Normal	Ollier’s disease, mild osteoporosis, non-union	Radiation, neurofibromatosis, osteogenesis imperfecta	Osteonecrosis, infection
Quality of soft tissue	Normal	Spastic, obese, muscular	Fibrotic, post-radiation, small open wound	Tissue necrosis, infection, large open wound
Medical problems and medications	None	Smoking, hypertension, rheumatoid arthritis or other systemic arthritis	Diabetes, haemophilia, sickle cell anaemia, mild immunosuppression, bone-inhibition medication	Moderate immunosuppression, anti-metabolic chemotherapy

This classifies the cases into 3 categories:

1. Mild; 0–6 points
2. Moderate; 7–11 points
3. Severe; >12 points

These scores were used to determine the level of difficulty of these cases as well as the possible relationship between a high score and complications. The outcome measure was based on a combination of function, alignment and need for further intervention: this is considered excellent if the patients were fully weight-bearing and pain-free on a mechanically aligned limb without need for further surgery; good if the patient required more surgery to achieve union; and poor if irreversible complications occurred.

No statistical analysis was performed as the numbers reported are small. Descriptive statistics are used.

## Results

The mean age of the patients was 32 years (range 22–39). There were 11 males and 7 female patients. The aetiology was divided into 18 post-traumatic causes and 6 development-related abnormalities. Distraction osteogenesis was used for limb lengthening in 7 cases, for the reconstruction of bone defects or non-unions in 10 cases and for deformity corrections in 7 cases. Patient data are summarized in Table 2.

**Table 2:** Patient data

Group	Case	Presenting problem	Management (ex-fix days)	Conversion (delay days)	Conversion	Outcome	Paley score
Plating delayed	1	Atrophic non-union humerus	TSF reconstruction (159)	TSF delay to ORIF (12)	Refracture, second debridement	Pin track sepsis	8
	3	Valgus deformity correction femur	TSF deformity correction (70)	TSF delay to ORIF (35)	Pin tracks infected debrided	Good	6
	4	Varus deformity correction femur	TSF deformity correction (70)	TSF delay to ORIF (35)		Good	6
	9	Septic non-union	Ilizarov, cement spacer, bone graft (238)	Ilizarov to ORIF (28)	Pin tracks curetted	Good	6
Plating acute	5	Lengthening femur defect 7 cm	Ilizarov—LRS lengthening (242)	LRS acute ORIF	Repeat debridement, bone graft and ORIF	Non-union	9
	6	Segmental fracture tibia mal/non-union	Ilizarov reconstruction (221)	Ilizarov to ORIF	Pin tracks excised	Good	7
	7	Bow leg deformity L	TSF and deformity correction (29)	TSF to ORIF	Pin tracks excised	Good	5
	8	Bow leg deformity R	TSF and deformity correction (29)	TSF to ORIF		Good	5
	11	Atrophic non-union femur	LRS, corticotomy, bone transport (266)	LRS to ORIF	Pin tracks excised	Good	6
	12	Non-union distal tibia	Ilizarov deformity correction (218)	Ilizarov to ORIF	Pin tracks curetted	Good	7
	15	Lengthening femur defect 5 cm	LRS, corticotomy (97)	LRS to ORIF	Distraction device	Good	6
	16	Lengthening femur defect 5 cm	LRS, corticotomy (91)	LRS to ORIF	Pin tracks excised	Good	6
	18	Bow leg deformity L	TSF and osteotomy deformity correction (33)	TSF to ORIF	Pin tracks curetted	Good	4
	19	Bow leg deformity R	TSF and osteotomy deformity correction (33)	TSF to ORIF	Pin tracks curetted	Good	4
	23	Segmental fracture tibia mal/non-union	TSF reconstruction	TSF to ORIF	Pin tracks curetted	Good	6
	24	Oligotrophic non-union tibia	TSF reconstruction	TSF to ORIF	Pin tracks curetted	Good	6
Nail delayed	14	Comminuted tibia fracture, distal 1/3	Ilizarov, corticotomy, lengthening (281)	Ilizarov to nail (4)	Pin tracks curetted	Good	6
Nail acute	2	GA III B tib fib, non-union, shortened 5 cm	TSF reconstruction and plastics (370)	TSF acute nail	Delayed amputation (142)	Amputation	9
	10	Septic non-union femur	LRS, corticotomy, bone transport (266)	LRS to nail	Bone transport 12 cm	Good	6
	13	Septic non-union distal tibia	Trulok, corticotomy, bone transport (126)	Trulok to nail	Pin tracks excised	Good	5
	17	Segmental tibial fracture	TSF reconstruction and plastics (90)	TSF acute nail	Pin tracks curetted	Good	5
	20	GA III B tibial fibula	TSF reconstruction and plastics (218)	TSF acute nail	Delayed amputation (93)	Amputation	7
	21	Open fracture radius	TSF reconstruction (61)	TSF acute nail	Pin tracks curetted	Good	5
	22	Open fracture ulna	TSF reconstruction (61)	TSF acute nail	Pin tracks curetted	Good	5

The reasons for conversion to internal fixation included dissatisfaction with the period in external fixation for 11 cases, persistent pin track infections in 8 cases, docking site-related problems in 4 cases and a refracture in one patient. The mean external fixator time was 185 days (61–370). Using the criteria described earlier, 20 limbs (83.3 %) had an excellent result, 2 patients had a good result (requiring further surgery to achieve union) and two with poor results (8.4 %).

**Fig. 1:**
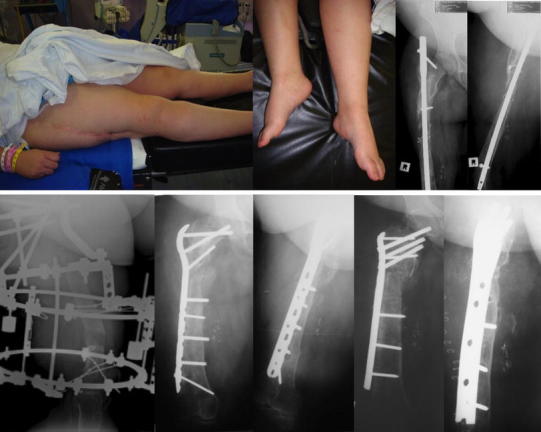
A 31-year-old female presented with a subtrochanteric non-union and a 12-cm leg length discrepancy after 14 previous surgeries. This reconstruction (Paley’s level of difficulty 9) required a second procedure (internal fixation and bone graft) to promote union after the initial conversion procedure (original frame time 242 days)

Both patients with poor results had requested amputations for persistent painful septic non-unions. These cases had prolonged frame time (280–370 days) and had high scores using Paley’s level of difficulty (7, 9) (Fig. 1).

Acute conversion was done in 19 limbs and delayed conversion in 5 of the 24 limbs. Although 17 limbs (89.4 %) in the acute conversion group had a good outcome (16 limbs of which with an excellent result), two limbs (10.6 %) had a poor result and required amputation. No deep infections were encountered in the acute conversion to plate fixation group. However, both amputations were after acute conversion to intramedullary nails after initial treatment for tibial septic non-unions. All cases in the delayed conversion group had a good outcome with the 4 limbs (80 %) having an excellent result. The number of cases in this group is small; the single delayed conversion to an intramedullary nail had no complications.

## Discussion

**Fig. 2:**
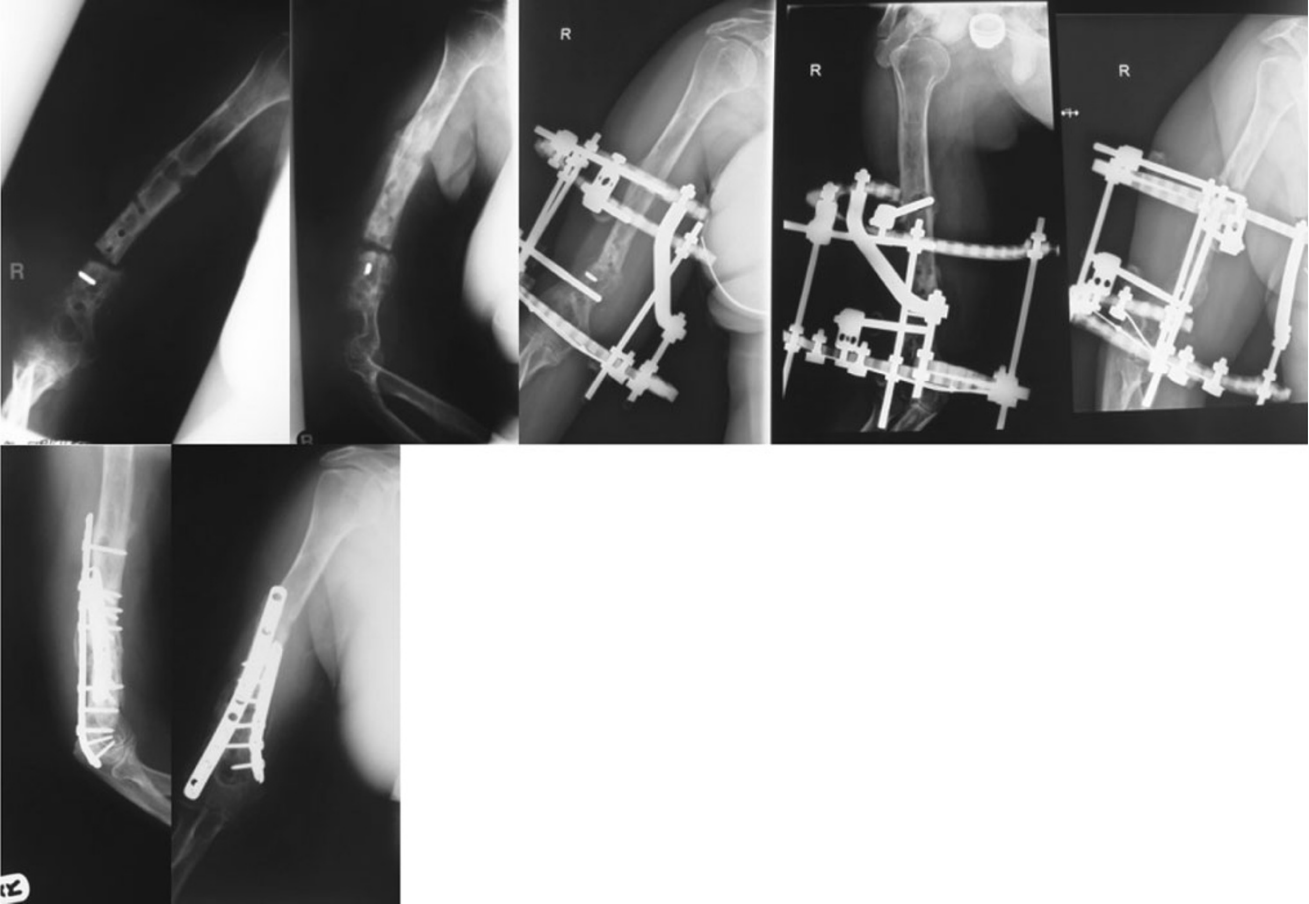
A 41-year-old female presented with an atrophic non-union of the humerus (Paley’s level of difficulty 8) which was managed with both Ilizarov and TSF frames (frame time 159 days) before being plated. The procedure was performed after a delay to allow secondary debridement for persistent pin track sepsis

This retrospective case series provides some support for the strategy of conversion from external to internal fixation. The number of complications was low, considering the severity of these cases, with an average Paley’s level of difficulty score of 6 (moderate). Plate fixation had a lower complication rate in the acute conversion group in comparison with intramedullary nails. This concurs with the findings of Rozbruch et al. [16, 17]. These authors also encountered a higher infection rate with the use of intramedullary nailing following external fixation lengthening (LATN) when compared to plating following lengthening (LAP). Our two amputations in this case series suggest that acute conversion to an intramedullary nail should be avoided when converting an external fixator to internal fixation if the original problem was a septic non-union. As to whether this risk is attenuated when there is a delay between fixator removal and nail introduction requires further study with a larger sample (Fig. 2).

Infection remains a problem during prolonged external fixation and is a risk when the method of fixation is changed to internal fixation. The average follow-up in this study was 20 months with a shorter minimum follow-up period; thus, the infection rates quoted in this case series have to be interpreted with some caution as occult sepsis may not be excluded conclusively. Another shortcoming in this study is that initial pin track infections prior to conversion were treated empirically and culture and sensitivity results unavailable. As both amputations were due to persistent infection, knowledge of pre- and post-conversion bacteriology may have provided further information in terms of risk factors and reasons for conversion failure.

The heterogeneity of patients in a reconstructive setting and the small sample in this case series makes it difficult to weigh the impact of medical comorbidities on outcome. We found the Paley level of difficulty score in femoral lengthening helpful as a system to quantify the additive nature of these negative effects. However, the system of scoring has to be validated further or be evolved to a more comprehensive limb reconstruction scoring system.

## Conclusion

Complex reconstruction surgery on limbs based on the technique of distraction osteogenesis will entail prolonged periods of external fixation. There will be, due to the nature of complexity of cases, a need for conversion to internal fixation owing to reasons of patient non-compliance, failure to progress in treatment or persistent complications with continued use of the external fixator device. This series supports the practice of conversion and identifies that plate devices appear to produce fewer deep sepsis complications, particularly when the original presenting diagnosis is a septic non-union.
